# Toward a comprehensive evidence map of overview of systematic review methods: paper 1—purpose, eligibility, search and data extraction

**DOI:** 10.1186/s13643-017-0617-1

**Published:** 2017-11-21

**Authors:** Carole Lunny, Sue E. Brennan, Steve McDonald, Joanne E. McKenzie

**Affiliations:** 1 Cochrane Australia, School of Public Health and Preventive Medicine, Monash University, Melbourne, Australia; 2 School of Public Health and Preventive Medicine, Monash University, 553 St Kilda Road, Melbourne, VIC 3004 Australia

**Keywords:** Overviews of systematic reviews, Overview, Meta-review, Umbrella review, Review of reviews, Overview methods, Systematic review methods, Evidence mapping, Evaluation of methods, Evidence synthesis

## Abstract

**Background:**

Overviews of systematic reviews attempt to systematically retrieve and summarise the results of multiple systematic reviews. Methods for conducting, interpreting and reporting overviews are in their infancy. To date, there has been no evidence map of the methods used in overviews, thus making it difficult to determine the gaps and priorities for methods research. Our objectives were to develop and populate a comprehensive framework of methods for conducting, interpreting and reporting overviews (stage I) and to create an evidence map by mapping studies that have evaluated overview methods to the framework (stage II).

**Methods:**

We searched methods collections (e.g. Cochrane Methodology Register, Meth4ReSyn library, AHRQ Effective Health Care Program) to identify eligible studies for both stages of this research. In stage I, cross-sectional studies, guidance documents and commentaries that described methods proposed for, or used in, overviews were used to develop and populate the framework of methods. Drafts and multiple iterations of the framework were discussed and refined by all authors. In stage II, we identified and described studies evaluating overview methods and mapped these evaluations to the framework.

**Results:**

In this paper, we present results for the four initial steps of conducting an overview: (a) specification of the purpose, objectives and scope, (b) specification of the eligibility criteria, (c) search methods and (d) data extraction. Twenty-nine studies mentioned or described methods relevant to one or more of these steps. In the developed framework, identified methods and approaches were grouped according to the steps an overview author would need to undertake. Fifteen studies evaluated identified methods, all of which mapped to the search methods step. These studies either reported the development and evaluation of a new search filter to retrieve systematic reviews or compared the performance of multiple filters.

**Conclusion:**

Gaps in the evaluation of methods were found for the majority of steps in the framework. More empirical studies are needed to evaluate the methods outlined and provide a comprehensive evidence map. The framework is useful for planning these evaluations and for planning methods required to deal with challenges that arise when conducting an overview.

**Electronic supplementary material:**

The online version of this article (10.1186/s13643-017-0617-1) contains supplementary material, which is available to authorized users.

## Background

Overviews of systematic reviews synthesise the results of multiple systematic reviews. Overviews are typically broader in scope than systematic reviews (SRs) and may examine different interventions for the same condition, the same intervention for different conditions, or the same intervention for the same condition but focusing on different outcomes [1–4].

The number of published overviews has increased steadily in recent years largely in response to the increasing number of SRs [5, 6]. The main steps and many of the methods used in the conduct of SRs are directly transferrable to overviews, such as independent study selection and data extraction [7]. However, many features are unique to overviews and require the application of different or additional methods. For example, methods for assessing the quality or the risk of bias of SRs, dealing with the inclusion of the same trial in multiple SRs, dealing with out-of-date SRs, and dealing with discordant results across SRs [6].

Despite the growth in overviews, there has been no evidence map identifying the range of methods for overviews and examining the evidence for using these methods. Evidence mapping is a systematic method used to characterise and catalogue a body of literature pertaining to evidence on a topic and is useful for identifying gaps in the literature [8, 9]. Evidence mapping has been commonly used to map the effects of healthcare interventions; however, the approach may also be applied for mapping the evidence on other topics, such as collating and synthesising evidence on the range and performance of research methods.

It is critical to determine whether there is evidence to support the use of methods for overviews because the validity and reliability of the findings from overviews depend on the performance of the underlying methods. This research aims to provide a comprehensive framework of overview methods and the evidence underpinning these methods—an evidence map of overview methods. In doing so, we aim to help overview authors plan for common scenarios encountered when conducting an overview and enable prioritisation of methods development and evaluation.

## Objectives

The objectives of this study were to (a) develop and populate a comprehensive framework of methods that have been used, or may be used, in conducting, interpreting and reporting overviews of systematic reviews of interventions (stage I); (b) map studies that have evaluated these methods to the framework (creating an evidence map of overview methods) (stage II); and (c) identify unique methodological challenges of overviews and methods proposed to address these.

**Fig. 1:**
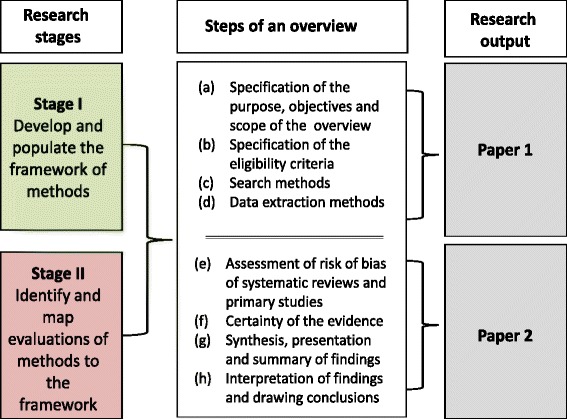
Summary of the research reported in each paper

This paper is the first of two companion papers. In this first paper, we present the methods framework for the four initial steps of conducting an overview: (a) specification of the purpose, objectives and scope of the overview; (b) specification of the eligibility criteria; (c) search methods and (d) data extraction methods (stage I). We then map studies evaluating methods to this framework (stage II). In a second paper, we will present the methods framework, and a map of evaluation studies, for the subsequent steps in conducting an overview: assessing risk of bias of primary studies and SRs; certainty of evidence arising from the overview; synthesis, presentation and summary of findings; and interpretation of findings and drawing conclusions (Fig. 1).

We use the term ‘methods framework’ (or equivalently, ‘framework of methods’) to describe the organising structure we have developed to group related methods and against which methods evaluations can be mapped. The highest level of this structure is the broad steps of conducting an overview (e.g. search methods). The methods framework, together with the studies that have evaluated these methods, form the evidence map of overview methods.

## Methods

**Fig. 2:**
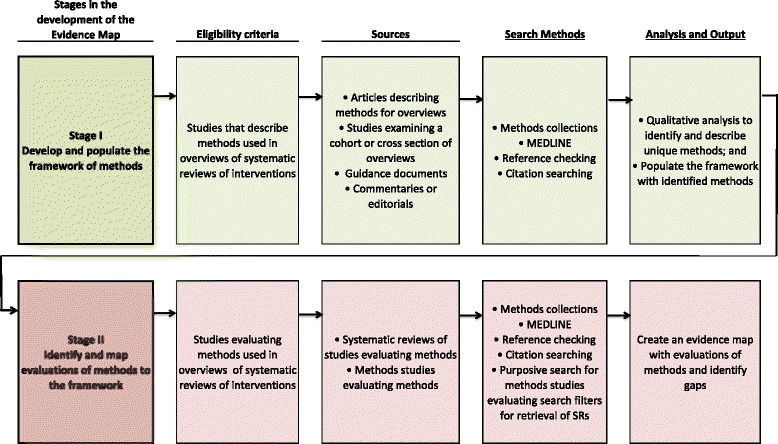
Stages in the development of an evidence map of overview methods

A protocol for this study has been published [10]. The methods for the two stages (Fig. 2) are now briefly described, along with deviations from the planned methods.

### Stage I: development and population of the framework of methods

#### Search methods

We searched MEDLINE from 2000 onwards and the following methods collections: Cochrane Methodology Register, Meth4ReSyn library, Scientific Resource Center Methods library of the AHRQ Effective Health Care Program, and Cochrane Colloquium abstracts. Searches were last run on December 2, 2015 (see Additional file 1 for search strategies). We also set aside any methods articles that we identified through screening citations as part of a related research project to develop a search strategy to identify overviews in MEDLINE [5]. To identify other potentially relevant studies, we examined the reference lists of included studies and undertook forward citation searches of seminal articles using Google Scholar, Scopus and Web of Science. We contacted authors of posters to retrieve the poster, or the full report of the study, and to ask if they were aware of any related methods articles. We planned to contact researchers with expertise in methods for overviews to identify articles missed by our search, but did not undertake this step due to time constraints.

#### Eligibility criteria

For the development and population of the framework, we identified articles describing methods used, or recommended for use, in overviews of systematic reviews of interventions.

Inclusion criteria:

i. Articles describing methods for overviews of systematic reviews of interventions
ii. Studies examining methods used in a cross-section or cohort of overviews
iii. Guidance (e.g. handbooks and guidelines) for undertaking overviews
iv. Commentaries or editorials that discuss methods for overviews

Exclusion criteria:

i. Articles published in languages other than English
ii. Studies describing methods for network meta-analysis
iii. Articles exclusively about methods for overviews of other review types (i.e. not of interventions)

We populated the framework with methods that were different or additional to those required to undertake a SR of primary research. Methods evaluated in the context of other ‘overview’ products, such as guidelines, which were of relevance to overviews, were included.

The eligibility criteria were piloted by three reviewers independently on a sample of articles retrieved from the search to ensure consistent application.

#### Study selection

Two reviewers independently reviewed titles and abstracts for their potential inclusion against the eligibility criteria. Full-text articles were retrieved when both reviewers agreed that inclusion criteria were met or when there was uncertainty. Any disagreement was resolved by discussion or by arbitration of a third reviewer. In instances where there was limited or incomplete information regarding a study’s eligibility (e.g. when only an abstract was available), the study authors were contacted to request the full text or further details.

#### Data extraction, coding and analysis

One author collected data from all included articles using a pre-tested form; a second author collected data from a 50% sample of the articles.

##### Data collected on the characteristics of included studies

We collected data about: (i) the type of article (coded as per our inclusion criteria), (ii) the main contribution(s) of the article (e.g. critique of methods), (iii) the extent to which each article described methods or approaches pertaining to each step of an overview (e.g. mention without description, described—insufficient detail to implement, described—implementable), (iv) a precis of the methods or approaches covered and (v) the data on which the article was based (e.g. audit of methods used in a sample of overviews, author’s experience).

##### Coding and analysis to develop and populate the framework of methods

We planned to code articles in NVivo software, applying a coding frame to extract descriptions of methods pertaining to each step of an overview [10]. However, during the initial phases of analysis, we found the extracts difficult to interpret when read out of context because many methods were either sparsely described or were inferred rather than explicit. As a consequence of the difficulty coding these data, we revised our analytic approach. We separated studies that described a method pertaining to a step in the overview process, from those that made cursory mention of a method. The subset of articles coded as providing description were read by two authors (CL and SB, JM or SM) who independently drafted the framework for that step to capture and categorise all identified or inferred methods. To ensure comprehensiveness of the framework, methods were inferred when a clear alternative existed to a reported method (e.g. using decision rules or an algorithm to combine eligibility criteria was rarely mentioned, but was clearly an option for multiple sub-steps).

The drafts and multiple iterations of the framework were discussed and refined by all authors, during which we delineated unique decision points faced when planning each step of an overview (e.g. determining eligibility criteria to deal with SRs with overlap, determining how discrepant data across SRs will be handled) and the methods/options available for each. We grouped conceptually similar approaches together and extracted examples to illustrate the options. For example, we categorised all approaches that involved specifying criteria to select one SR from multiple overlapping SRs together, and then listed examples of criteria suggested in included studies (e.g. select most recent SR, highest quality, most comprehensive).

### Stage II: Identification and mapping of evaluations of methods

#### Search methods

In addition to the main searches outlined in the  'Search methods' section for Stage I, we planned to undertake purposive searches to locate evaluations of methods where the main searches were unlikely to have located these evaluations. For this paper, we undertook a purposive search to locate evaluations of search filters for the retrieval of SRs (Additional file 2) since articles describing the development and evaluation of search strategies for SRs may reasonably not have mentioned ‘overviews’ (or its synonyms) and thus would not be identified in the main searches. For the other steps, the identified methods were specific to overviews, so evaluations were judged likely to be retrieved by our main search.

#### Eligibility criteria

To create the evidence map, we identified articles describing evaluations of methods for overviews of systematic reviews of interventions.

Inclusion criteria:

i. SRs of methods studies that have evaluated methods for overviews
ii. Methods studies that have evaluated methods for overviews

Exclusion criteria:

i. Articles published in languages other than English
ii. Methods studies that have evaluated methods for network meta-analysis

We added the additional criterion that methods studies had to have a stated aim to evaluate methods, since our focus was on evaluation and not just application of a method.

#### Study selection

We used the same process for determining which studies met the inclusion criteria for stage II as for stage I (‘Study selection’ section Stage I).

#### Data extraction

The only methods evaluations identified were evaluations of search filters for SRs, from which we extracted the data listed in Table 1. We had originally planned to extract quantitative results from the methods evaluations relating to the primary objectives; however, on reflection, we opted not to do this since we felt this lay outside the purpose of the evidence map. Data were extracted independently by two authors (CL, JEM) from four (of 15) studies. The remaining data were extracted by one author (CL).

**Table 1:** Data extracted from methods studies evaluating search filters for SRs

Data extracted	Description
Study characteristics	Citation details
	Primary objective
Search filter evaluation details	Type of search filter evaluation (categorised as single search filter evaluation, comparative search filter evaluation, comparative database evaluation)
	Health field filter designed for
	Number of filters evaluated
	Number of filters developed by author
	Databases filters tested in and the interface(s)
	Technique to identify and/or create gold standard
	Sample size of the gold standard set or validation set
	Performance measures (e.g. sensitivity/recall, specificity)
	Search dates of the gold standard or validation set
	Name of filters evaluated
Risk of bias criteria	Existence of a protocol
	Validation on a data set distinct from the derivation set

#### Assessment of the risk of bias

We planned to report the characteristics of the stage II evaluation studies that may plausibly be associated with bias. For methods evaluations of search filters for identifying SRs, we used assessment criteria informed by Harbour [11]. The assessment criteria included existence of a protocol and validation of the filter on a data set distinct from the derivation set (external validation).

#### Analysis

The yield and characteristics of the methods evaluation studies were described and mapped to the framework of methods.

## Results

### Results of the search

**Fig. 3:**
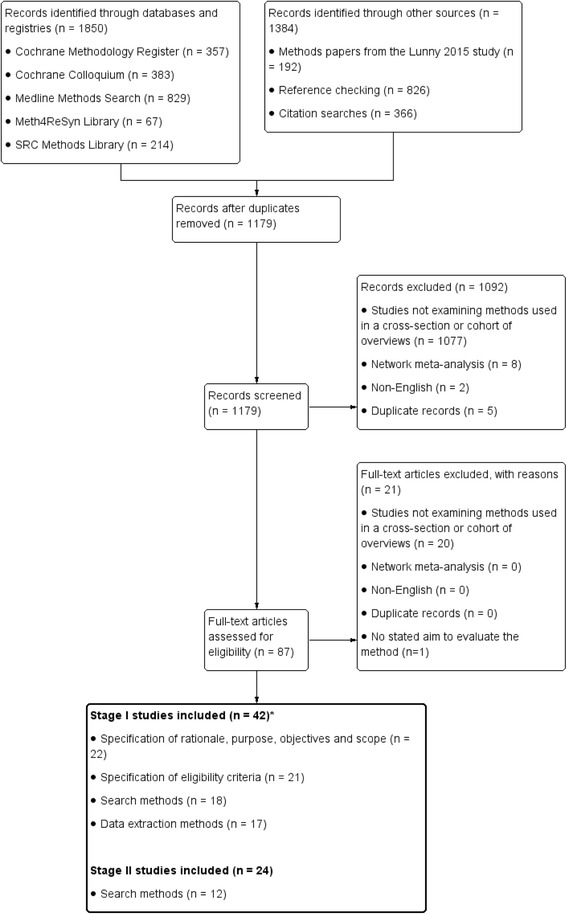
Flowchart of studies retrieved for both stages I and II. *The 42 stage I studies contributed to multiple steps

We retrieved 1850 records through searching databases and methods collections. A further 1384 records were identified through other sources (methods articles identified as part of a related research project [5], reference checking, and forward citation searching). After removal of duplicate records, 1179 records remained (Fig. 3). From screening titles and abstracts, we excluded 1092 records that were ineligible. We assessed 87 full-text reports for eligibility and excluded 21, with reasons noted in Additional file 3. Of the remaining 66, 42 were included in stage I and 24 in stage II.

**Fig. 4:**
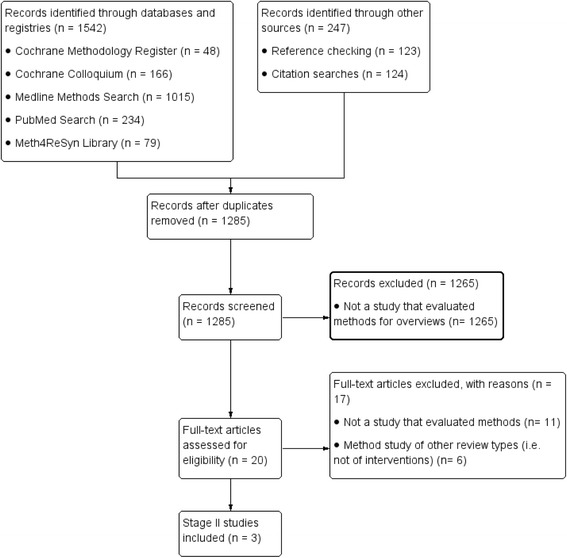
Flowchart of stage II studies of search filter evaluations

Our purposive search strategy (dated May 2016) to identify studies evaluating search filters for the retrieval of SRs resulted in the inclusion of three more stage II studies (see Fig. 4 for details), bringing the total number of methods evaluations to 27.

Of the 42 stage I and 27 stage II studies, 29 and 15, respectively, pertained to one or more of the four initial steps in conducting an overview and so are included in this first paper; the remainder will be included in our second companion paper. All 15 stage II studies were evaluations of search filter studies for retrieval of SRs.

### Stage I: development and population of the framework of methods

We first describe the characteristics of the included articles (see ‘Characteristics of included articles’; Table 2), followed by presentation of the developed methods framework. This presentation is organised into sections representing the broad steps of conducting an overview (sections ‘Specification of purpose, objectives and scope’, ‘Specification of eligibility criteria’, ‘Search methods’ and ‘Data extraction’; Tables 3, 4, 5 and 6). In each section, we orient readers to the structure of the methods framework, which includes a set of steps and sub-steps (e.g. under ‘Search methods’, the steps are ‘plan the sources to search’, ‘plan the search strategy for retrieval of SRs’, and ‘plan how primary studies will be retrieved’). Components within the tables are referred to using labels and numbers (e.g. 2.1.3). We highlight methods/approaches to deal with commonly encountered scenarios for which overview authors need to plan (see ‘Addressing common scenarios unique to overviews’; Table 7). Our description is focused on methods/options that are distinct, have added complexity, compared with SRs of primary studies, or have been proposed to deal with major challenges in undertaking an overview. Importantly, the methods/approaches and options reflect the ideas presented in the literature and should not be interpreted as endorsement for the use of the methods. Reporting considerations for all steps are reported in Additional file 4.

**Table 2:** Characteristics of stage I descriptive studies

			Steps in the conduct of an overview			
Citation	Type of study	Summary description of the article	Purpose, objectives, scope	Eligibility criteria	Search methods	Data extraction
**Baker 2014** [29] The benefits and challenges of conducting an overview of systematic reviews in public health: a focus on physical activity.	Article describing methods for overviews	• Describes the usefulness of overviews for decision makers and summarises some procedural steps to be undertaken • Provides a case study of an overview on public health interventions for increasing physical activity	✓✓	✓✓	✓✓	
**Becker 2008** [1] Overviews of reviews.	Guidance for undertaking overviews	• Early guidance providing the structure and procedural steps for the production of an overview • Details the different purposes of an overview, providing examples and describes how to present findings through tables and figures	✓✓	✓✓	✓✓	✓✓
**Bolland 2014** [30] A case study of discordant overlapping meta-analyses: vitamin D supplements and fracture.	Article describing methods for overviews	• Describes criteria for explaining differences in overlapping M-As with discordant conclusions • Builds on the guide to interpret discordant SRs proposed by Jadad 1997 • Suggests reporting items there are overlapping trials in M-As	✓	✓✓		✓✓
**Caird 2015** [31] Mediating policy-relevant evidence at speed: are systematic reviews of systematic reviews a useful approach?	Article describing methods for overviews	• Describes the methodological challenges in the production of overviews that mediate existing synthesised knowledge to policy makers • Describes the trade-offs between producing a rapid overview and its comprehensiveness and reliability	✓✓	✓✓	✓✓	✓✓
**Chen 2014** [2] Scientific hypotheses can be tested by comparing the effects of one treatment over many diseases in a systematic review.	Study examining methods used in a cohort of overviews	• Identifies possible aims of an overview as being to detect unintended effects, improve the precision of effect estimates, or explore heterogeneity of effect across disease groups • Describes the value and pitfalls of synthesis of M-As using three case studies	✓✓	✓✓		
**CMIMG 2012** [4] Review type and methodological considerations.	Guidance for undertaking overviews	• Provides updated Cochrane guidance on the purpose and conduct of overviews • Builds on the Cochrane guidance for overviews by Becker 2008 • Describes the factors in the decision to conduct an overview vs. an SR	✓✓	✓✓	✓✓	✓✓
**Cooper 2012** [32] The overview of reviews: unique challenges and opportunities when research syntheses are the principal elements of new integrative scholarship.	Article describing methods for overviews	• Describes steps in the conduct of an overview and methods to address challenges (for example dealing with overlap in primary studies) • Describes methods for second order meta-analysis	✓✓	✓✓	✓✓	✓✓
**Flodgren 2011** [33] Challenges facing reviewers preparing overviews of reviews.^a^	Article describing methods for overviews	• Mentions the issue of missing or inadequately reported data • Mentions the challenges in summarising and evaluating large amounts of heterogeneous data				✓
**Foisy 2011** [34] Mixing with the ‘unclean’: Including non-Cochrane reviews alongside Cochrane reviews in overviews of reviews.^a^	Article describing methods for overviews	• Describes some challenges inherent in the eligibility criteria process (defining AMSTAR scoring as inclusion criteria, inclusion of non-Cochrane reviews alongside Cochrane reviews) • Develops inclusion criteria to minimise overlap in primary studies		✓✓		✓✓
**Hartling 2012** [35] A descriptive analysis of overviews of reviews published between 2000 and 2011.	Study examining methods used in a cohort of overviews	• Describes steps in the conduct of overviews and methods used • Describes methodological standards for SRs (MECIR) and their applicability to overviews • Describes PRISMA reporting standards and their applicability to overviews	✓	✓✓	✓✓	✓✓
**Hartling 2013** [36] Generating empirical evidence to support methods for overviews of reviews.^a^	Study examining methods used in a cohort of overviews	• Mentions challenges relating to the eligibility criteria process in terms of SR quality, search dates, the strength of the evidence to include, etc.	✓	✓		
**Hartling 2014** [37] Systematic reviews, overviews of reviews and comparative effectiveness reviews: a discussion of approaches to knowledge synthesis.	Article describing methods for overviews	• Briefly defines overviews, mentions the purposes in conducting an overview, and discusses some methodological challenges	✓			
**Ioannidis 2009** [38] Integration of evidence from multiple meta-analyses: a primer on umbrella reviews, treatment networks and multiple treatments meta-analyses.	Article describing methods for overviews	• Defines umbrella reviews as a pre-step to network meta-analysis • Describes challenges of overviews and a checklist of pitfalls	✓✓			
**James 2014** [39] Informing the methods for public health overview reviews: a descriptive analysis of Cochrane and non-Cochrane public health overviews.^a^	Study examining methods used in a cohort of overviews	• Briefly describes several steps in the conduct of overviews including determining the eligibility criteria and search methods • Compares Cochrane and non-Cochrane reviews in terms of restrictions on inclusion criteria	✓	✓	✓	
**Joanna Briggs Institute (JBI) 2014** [40, 41] Methodology for JBI umbrella reviews.	Guidance for undertaking overviews	• Provides guidance as to what methods should be used at which step in the conduct of an overview • Provides stylistic conventions for overviews to meet publication and reporting criteria for the JBI Database of Systematic Reviews and Implementation Reports	✓✓	✓✓	✓✓	✓✓
**Kovacs 2014** [42] Overviews should meet the methodological standards of systematic reviews.	Commentary or editorial that discuss methods for overviews	• Mentions four methodological shortcomings of one overview on surgical interventions as a letter to the editor				✓
**Kramer 2009** [43] Preparing an overview of reviews: lessons learned.^a^	Article describing methods for overviews	• Mentions the challenges encountered when the authors conducted three overviews including missing information when extracting data		✓		✓
**Li 2012** [44] Quality and transparency of overviews of systematic reviews.	Article describing methods for overviews	• Presents a pilot reporting/quality checklist • Evaluates a cohort of overviews using the pilot tool, with the mean number of items but no details of the items	✓	✓	✓	✓
**Pieper 2012** [6, 45] Overviews of reviews often have limited rigor: a systematic review.	Study examining methods used in a cohort of overviews	• Describes the methods used in a cohort of overviews • Recommends using validated search filters for retrieval of SRs • Discusses whether to update the overview by including primary studies published after the most recent SR	✓	✓✓	✓✓	✓
**Pieper 2014** [46] Methodological approaches in conducting overviews: current state in HTA agencies.	Article describing methods for overviews	• Describes the methods recommended in 8 HTA guideline documents related to overviews • Compares the Cochrane Handbook guidance to guidance produced by HTA agencies		✓	✓	
**Pieper 2014** [47] Up-to-dateness of reviews is often neglected in overviews: a systematic review.	Study examining methods used in a cohort of overviews	• Describes the process of searching for primary studies in an overview • Presents decision rules for when to search for primary studies • Outlines search methods in terms of sequential versus parallel searching for SRs and primary studies			✓✓	
**Robinson 2016** [48–52] Integrating bodies of evidence: existing systematic reviews and primary studies.	Article describing methods for overviews	• Describes the steps to undertake a complex review that includes multiple SRs, which is similar to overviews • Discusses challenges inherent in the production of complex reviews that include SRs	✓✓	✓✓	✓✓	✓✓
**Ryan 2005** [53, 54] Building blocks for meta-synthesis: data integration tables for summarising, mapping, and synthesising evidence on interventions for communicating with health consumers.	Article describing methods for overviews	• Presents tabular methods to deal with the preparation of overview evidence • Discusses the data extraction process and organisation of data • Presents a table of taxonomy of outcomes from the included SRs, and a data extraction table based on this taxonomy	✓✓			✓✓
**Salanti 2011** [3] Evolution of Cochrane intervention reviews and overviews of reviews to better accommodate comparisons among multiple interventions.	Guidance for undertaking overviews	• Provides Cochrane guidance on the definition of an overviews and as compared to SRs • Suggests broadening the search in an overview to include individual studies • Suggests missing data should be retrieved from original reports	✓✓	✓✓	✓✓	✓
**Silva 2015** [55] Overview of systematic reviews - a new type of study.	Study examining methods used in a cohort of overviews	• Examines a cohort of Cochrane reviews for methods used • Documented the sources and types of search strategies conducted			✓✓	
**Singh 2012** [56] Development of the Metareview Assessment of Reporting Quality (MARQ) Checklist.	Article describing methods for overviews	• Presents a pilot reporting/quality checklist • Evaluates four case studies using the pilot tool, with the mean number of items but no details of the items	✓✓		✓✓	
**Smith 2011** [57] Methodology in conducting a systematic review of systematic reviews of healthcare interventions.	Article describing methods for overviews	• Describes some steps and challenges in undertaking an overview, namely search methods, study selection, quality assessment, and presentation of results • Presents tabular methods for the preparation of an overview	✓✓	✓✓	✓✓	✓
**Thomson 2010** [58] The evolution of a new publication type: Steps and challenges of producing overviews of reviews.	Article describing methods for overviews	• Describes some steps in undertaking an overview and the challenges inherent in production of overviews • Discusses that gaps or lack of currency in included evidence will weaken the overview findings	✓✓	✓✓		
**Thomson 2013** [59] Overview of reviews in child health: evidence synthesis and the knowledge base for a specific population.	Study examining methods used in a cohort of overviews	• Describes the process of including trials in overviews ▪ Discusses the challenge of overview topics differing from the topics of the included SRs ▪ Provides potential solutions as to what to do when mixed populations are reported in SRs and how to extract age subgroup data	✓✓	✓✓	✓✓	

*AMSTAR* A MeaSurement Tool to Assess systematic Reviews, *CMIMG* Comparing Multiple Interventions Methods Group, *JBI* Joanna Briggs Institute, *PRISMA* Preferred Reporting Items for Systematic reviews and Meta-Analyses, *HTA* health technology assessment, *MECIR* Methodological Expectations of Cochrane Intervention Reviews, *SR* systematic review, *M-As* meta-analyses

^a^Indicates a poster presentation

✓✓ Indicates a study describing one or more methods

✓ Indicates a study mentioning one or more methods

**Table 3:** Specification of purpose, objectives and scope

Step	Sub-step	Methods/approaches	Sources ▪ Examples
1.0 Determine stakeholder involvement in planning the overview			
	1.1 Agree on who is responsible for setting the overall purpose and objectives		
		1.1.1 Commissioners of the overview	Whitlock 2008 [48–52]
		1.1.2 Researcher or author team	Becker 2008 [1]; Whitlock 2008 [48–52]
		1.1.3 Multiple/all stakeholders in collaboration	Caird 2015 [31]; Cooper 2012 [32]; Hartling 2012 [35]; JBI 2015 [40, 41]; Ryan 2009 [53, 54]; Whitlock 2008 [48–52]
	1.2 Determine the extent and approach to stakeholder involvement in defining the purpose, objectives and scope of the overview (i.e. who, on what aspects, at what stage(s), how)		Caird 2015 [31]; Hartling 2012 [35]
2.0 Define the purpose, objectives and scope			
	2.1 Define the purpose of the overview		
		2.1.1 Map the type and quantity of available evidence (e.g. types of interventions, outcomes, populations/settings, study designs but not effects)	Becker 2008 [1]; Caird 2015 [31]; CMIMG 2012 [4]; Cooper 2012 [32]; Hartling 2014 [37]; Salanti 2011 [3]
		2.1.2 Compare multiple interventions with the intent of drawing inferences about the comparative effectiveness of the interventions intervention for the same condition, problem or population	Becker 2008 [1]; CMIMG 2012 [4]; Cooper 2012 [32]; Hartling 2012 [35]; Hartling 2014 [37]; Ioannidis 2009 [38]; Ryan 2009 [53, 54]; Salanti 2011 [3]; Smith 2011 [57] ▪ An overview of interventions for nocturnal enuresis (Becker 2008 [1])
		2.1.3 Summarise the effects of an intervention for the same condition, problem or population where different outcomes are addressed in different SRs	Becker 2008 [1]; CMIMG 2012 [4]; Cooper 2012 [32]; Hartling 2012 [35]; Hartling 2014 [37]; Ryan 2009 [53, 54]; Salanti 2011 [3]; Smith 2011 [57] ▪ An overview of hormone replacement therapy for menopause where outcomes may include bone density, menopausal symptoms, cardiovascular risk/ events, cognitive function etc. (Becker 2008 [1])
		2.1.4 Summarise the effects of an intervention across conditions, problems or populations (e.g. “borrowing strength” when there is sparse data for a single condition and a similar mechanism of action for the intervention is predicted across conditions)	Becker 2008 [1]; Chen 2014 [2]; CMIMG 2012 [4]; Cooper 2012 [32]; Hartling 2012 [35]; Hartling 2014 [37]; Ryan 2009 [53, 54]; Salanti 2011 [3]; Smith 2011 [57] ▪ An overview of vitamin A for different populations and conditions (Becker 2008 [1])
		2.1.5 Summarise unexpected (including adverse) effects of an intervention across conditions, problems or populations	Becker 2008 [1]; Chen 2014 [2]; CMIMG 2012 [4]; Cooper 2012 [32]; Hartling 2012 [35]; Ioannidis 2009 [38]; Salanti 2011 [3]; Smith 2011 [57] ▪ An overview of adverse effects of NSAIDs when used for osteoarthritis or rheumatoid arthritis or menorrhagia (Becker 2008 [1])
		2.1.6 Identify and explore reasons for heterogeneity in the effects of an intervention (e.g. by examining reasons for discordant results or conclusions across SRs)	Bolland 2014 [30]; Caird 2015 [31]; Chen 2014 [2]; Cooper 2012 [32]; JBI 2015 [40, 41]; Singh 2012 [56]; Smith 2011 [57] ▪ Overview investigating differences between the meta-analyses of vitamin D for prevention of fracture (Bolland 2014 [30])
		2.1.7 Other purposes	CMIMG 2012 [4]; Cooper 2012 [32]; Hartling 2014 [37]; JBI 2015 [40, 41]; Pieper 2012 [6, 45]; Robinson 2015 [48–52]; Ryan 2009 [53, 54]
	2.2 Confirm that an overview is the appropriate type of study for addressing the purpose and objectives, as opposed to other types of reviews (i.e. intervention review, network meta-analysis)		
		2.2.1 Use a decision algorithm	Becker 2008 [1]; CMIMG 2012 [4]; Salanti 2011 [3] ▪ CMIMG editorial decision tree which covers decision points for choosing between an overview or a new or updated SR (with or without network meta-analysis) (Salanti 2011 [3])
		2.2.2 Use other reasoning (triggers), for example, a new or updated SR might be more appropriate than an overview when SRs: (i) are not available, or have insufficient overlap with the overview question/PICO, (ii) have methodological shortcomings (including not being up-to-date), (iii) are discordant and the reason for discordance cannot be identified (e.g. by methodological differences), and (iv) need independent confirmation (or disconfirmation) (e.g. where SR authors have conflicts of interest such as industry ties or funding)	Chen 2014 [2]; Hartling 2014 [37]; Whitlock 2008 [48–52]; Singh 2012 [56]; Smith 2011 [57]
	2.3 Determine any constraints that will restrict the scope of the overview (e.g. time, staffing, skill set)		Caird 2015 [31]; Cooper 2012 [32]; Pieper 2012 [6, 45]; Smith 2011 [57]
	2.4 Define the scope of the overview taking into account 2.1–2.3		
		2.4.1 Narrow scope-based on a well-defined question (specific PICOs) or methodological criteria restrictions (i.e. date range of eligible literature, sources searched, publication types and study designs, extent and quality of data extracted, type of synthesis undertaken)	Baker 2014 [29]; Chen 2014 [2]; CMIMG 2012 [4]; Cooper 2012 [32]; JBI 2015 [40, 41]; Pieper 2012 [6, 45]; Ryan 2009 [53, 54]; Salanti 2011 [3]; Thomson 2010 [58] ▪ Interventions restricted to a specific intervention for a specific condition/population (e.g. smoking cessation therapies for reducing harmful effects of smoking during pregnancy)
		2.4.2 Broad scope - based on a broadly defined question with diverse and multiple PICOs elements, or no methodological restrictions	Baker 2014 [29]; Caird 2015 [31]; Chen 2014 [2]; CMIMG 2012 [4]; Cooper 2012 [32]; JBI 2015 [40, 41]; Pieper 2012 [6, 45]; Pieper 2014 [46]; Ryan 2009 [53, 54]; Salanti 2011 [3]; Smith 2011 [57]; Thomson 2010 [58] ▪ Interventions of broad policy relevance (e.g. any intervention to reduce the harmful effects of smoking, including cessation therapies, mass media, and pricing policies.)
	2.5 Define the objectives using PICO elements (or equivalent) to develop an answerable question		Baker 2014 [29]; Becker 2008 [1]; Cooper 2012 [32]; Hartling 2012 [35]; JBI 2015 [40, 41]; Li 2012 [44]; Ryan 2009 [53, 54]; Smith 2011 [57]; Robinson 2015 [48–52]; Thomson 2010 [58]

*CMIMG* Comparing Multiple Interventions Methods Group, *JBI* Joanna Briggs Institute, *NSAIDs* nonsteroidal anti-inflammatory drugs, *PICOs* Population, Intervention, Comparison, Outcome, and Study design, *RoB* risk of bias, *SRs* systematic reviews

**Table 4:** Specification of eligibility criteria

Step	Sub-step	Methods/approaches	Sources ▪ Examples
1.0 Plan the eligibility criteria			
	1.1 Determine PICO eligibility criteria for the overview (and setting and timing if applicable)		Becker 2008 [1]; Cooper 2012 [32]; Ioannidis 2009 [38]; JBI 2015 [40, 41]; Li 2012 [44]; Thomson 2013 [59]
	1.2 Determine PICO eligibility criteria for SRs		
		1.2.1 Select only SRs that are similar (or narrower) in scope to the overview PICO elements (i.e. exclude SRs that include out-of-scope interventions/populations in addition to the intervention/population addressed by the overview)	Becker 2008 [1]; Cooper 2012 [32]; Foisy 2011 [34]; JBI 2015 [40, 41]; Robinson 2016 [48–52]; Ryan 2009 [53, 54]; Thompson 2013 [59]
		1.2.2 Select all SRs that address the PICO elements, including those broader in scope than the overview (i.e. SRs that include the intervention/ population addressed by the overview, plus other out-of-scope interventions/ populations). This may involve selecting: (i) any SR, irrespective of whether separate data are available for the subgroup of interest or (ii) limiting to SRs that present separate data for the subgroup of interest	Becker 2008 [1]; Cooper 2012 [32]; Kramer 2009 [43]; Ryan 2009 [53, 54]; Thompson 2013 [59]; Whitlock 2008 [48–52]
	1.3 Determine criteria (mechanisms) to select outcomes where there are multiple		
		1.3.1 Include all outcomes reported in included SRs	Becker 2008 [1]; Hartling 2012 [35]; Ryan 2009 [53, 54]; Thomson 2013 [59] ▪ Map the outcomes to a taxonomy (Ryan 2009 [53, 54])
		1.3.2 Select one or more outcomes using pre-specified criteria, for example: (i) outcomes judged important by subject specialists (e.g. consumers, policy makers), (ii) primary outcomes, and (iii) outcomes common to more than one SR	Caird 2015 [31]; Hartling 2014 [37]; Ioannidis 2009 [38]; JBI 2015 [40, 41]; Smith 2011 [57]; Thomson 2013 [59] ▪ Report only those outcomes common to more than one SR (Caird 2015 [31]; Hartling 2014 [37])
		1.3.3 Select one or more outcomes using pre-specified decision rules (e.g. combine selection criteria in an algorithm)	Inferred method
	1.4 Determine methodological eligibility criteria for SRs		
		1.4.1 Include all SRs that meet the PICO criteria (i.e. no methodological criteria applied)	Caird 2015 [31]
		1.4.2 Select SRs that meet minimum quality criteria or take a particular methodological approach. Minimum criteria include: (i) meets definition of an SR, (e.g. explicit search) (ii) up-to-date (iii) quality of the SR (e.g. based on selected criteria; cutoffs derived from AMSTAR score) (iv) use of best practice methods (e.g. specific RoB tools; Cochrane or AHRQ’s EPC methods) (v) free of conflicts of interest (e.g. no industry funding) (vi) reports sufficient primary study characteristics to interpret results (e.g. PICO elements, RoB assessment) Methodological approaches include: (vii) type of included primary studies (viii) type of data (ix) type of synthesis (e.g. meta-analysis, narrative)	Becker 2008 [1]; Chen 2014 [2]; Cooper 2012 [32]; Foisy 2011 [34]; Hartling 2013 [36]; James 2014 [39]; JBI 2015 [40, 41]; Robinson 2016 [48–52]; Smith 2011 [57]; Thompson 2013 [59]
	1.5 Determine eligibility criteria to deal with SRs with overlap		
		1.5.1 Include all SRs that meet the PICO, irrespective of overlap	Cooper 2012 [32]; Whitlock 2008 [48–52]
		1.5.2 Select one SR from multiple addressing the same question using pre-specified methodological criteria as outlined in 1.4.2	Cooper 2012 [32]; Pieper 2014 [46]; Robinson 2015 [48–52] ▪ Select the highest quality SR (Cooper 2012 [32])
		1.5.3 Select one SR from multiple addressing the same question using pre-specified decision rules (e.g. combine one or more eligibility criteria in an algorithm)	Cooper 2012 [32] ▪ Select the SR with the most complete information, and if these are equivalent, the M-A with the greatest number of primary studies (Cooper 2012 [32])
		1.5.4 Exclude SRs that do not contain any unique primary studies, when there are multiple SRs	Pieper 2014 [46]
	1.6 Determine whether to consider additional primary studies for inclusion		
		1.6.1 Do not include primary studies	Becker 2008 [1]; Caird 2015 [31]; Thompson 2013 [59]; Whitlock 2008 [48–52]
		1.6.2 Include primary studies if pre-specified eligibility criteria are met, for example: (i) when a SR is not up-to-date, (ii) when a SR is inconclusive (i.e. new studies may overturn the findings of a SR), (iii) when the included SRs provide incomplete coverage of evidence in relation to the overview PICO (e.g. missing one or more interventions, population subgroup, study design), and (iv) when there are concerns about the methods SRs used to identify and select studies	Baker 2014 [29]; Caird 2015 [31]; Cooper 2012 [32]; Pieper 2014 [46]; 2014 [47]; Thompson 2013 [59]; White 2009 [48–52] ▪ Include primary studies if the evidence in the SRs is inconclusive (e.g. when addition of a new primary study may overturn the findings) (Pieper 2014 [47]) ▪ Include primary studies if the SRs are assessed as low quality (Pieper 2014 [47])
		1.6.3 Include primary studies using pre-specified decision rules to determine eligibility (e.g. combine one or more eligibility criteria in an algorithm for selection)	Pieper 2014 [47]
2.0 Plan the study selection process			
	2.1 Determine the number of overview authors required to select studies^a^		
		2.1.1 Independent screening all stages by 2 or more authors	Becker 2008 [1]; Chen 2014 [2]; Hartling 2012 [35]; Li 2012 [44]; Pieper 2012 [6, 45]; 2014 [47]; Smith 2011 [57]
		2.1.2 One author screening at all stages	Hartling 2012 [35]; Li 2012 [44]; Pieper 2014 [47]
		2.1.3 One author screening titles/abstracts, 2 or more screening full text	Hartling 2012 [35]
		2.1.4 One screened at all stages, 2nd confirmed	Hartling 2012 [35]
		2.1.5 One screened at all stages, 2nd confirms if uncertainty	Hartling 2012 [35]

*AHRQ’s EPC* Agency for Healthcare Research and Quality ‘s Evidence-based Practice Center, *AMSTAR* A MeaSurement Tool to Assess systematic Reviews, *CMIMG* Comparing Multiple Interventions Methods Group, *JBI* Joanna Briggs Institute, *PICOs* Population, Intervention, Comparison, Outcome, and Study design, *RCT* randomised controlled trial, *SRs* systematic reviews

^a^Adaption of the step from SRs to overviews. No methods evaluation required, but special consideration needs to be given to unique issues that arise in conducting overviews

**Table 5:** Search methods

Step	Sub-step	Methods/approaches	Sources ▪ Examples
1.0 Plan the sources to search			
	1.1 Determine the type of sources to search		
		1.1.1 Select the types of databases to search (e.g. SR databases (e.g. Cochrane, Epistemonikos), prospective SR registers (e.g. PROSPERO), or general bibliographic databases (e.g. EMBASE, PubMed), or grey literature databases (e.g. conference databases, government websites))	Becker 2008 [1]; Baker 2014 [29]; Caird 2015 [31]; Cooper 2012 [32]; Hartling 2012 [35]; 2014 [37]; James 2014 [39]; JBI 2015 [40, 41]; Li 2012 [44]; Pieper 2012 [6, 45]; Pieper 2014 [47]; Silva 2015 [55]; Smith 2011 [57]; Thomson 2013 [59]; Whitlock 2008 [49–51]
		1.1.2 Select other types of sources (e.g. reference checking, forward citation searching, handsearching key journals)^a^	Cooper 2012 [32]; Hartling 2012 [35]; JBI 2015 [40, 41]; Li 2012 [44]; Smith 2011 [57]
		1.1.3 Select a combination of 1.1.1–1.1.2	Cooper 2012 [32]; Hartling 2012 [35]; JBI 2015 [40, 41]; Silva 2015 [55]; Smith 2011 [57]; Whitlock 2008 [48–52]
2.0 Plan the search strategy for retrieval of SRs			
	2.1 Determine the search filter to use in general databases		
		2.1.1 Select a published SR filter (e.g. EMBASE, MEDLINE, PubMed)	Cooper 2012 [32]; CMIMG 2012 [4]; JBI 2015 [40, 41]; Robinson 2016 [48–52]; Smith 2011 [57] ▪ Montori 2006 SR filter (Cooper 2012 [32])
		2.1.2 Develop a new search filter based on a conceptual approach or a textual analysis approach	Baker 2014 [29]; Caird 2015 [31]; Cooper 2012 [32]; Hartling 2012 [35]; JBI 2015 [40, 41]
3.0 Plan how primary studies will be retrieved, if eligibility criteria determines that primary studies should be included			
	3.1 Determine the sequence for searching		
		3.1.1 Run a parallel search strategy for both SRs and primary studies simultaneously	Baker 2014 [29]; JBI 2015 [40, 41]; Pieper 2014 [46]; Pieper 2014 [47]; Salanti 2011 [3]; Thomson 2013 [59]; Whitlock 2008 [49–51]
		3.1.2 Run a sequential search strategy first for SRs and second for primary studies (i.e. either develop a strategy to search for primary studies, or use the search strategies of the included SRs to search for primary studies)	Pieper 2014 [47]
	3.2 Use pragmatic/expedient approaches to retrieve primary studies		Caird 2015 [31] ▪ Consult experts (Caird 2015 [31])
	3.3 Select a combination of 3.1–3.2		

*CMIMG* Comparing Multiple Interventions Methods Group, *JBI* Joanna Briggs Institute, *PROSPERO* International Prospective Register of Systematic Reviews, *SRs* systematic reviews

^a^Adaption of the step from SRs to overviews. No methods evaluation required, but special consideration needs to be given to unique issues that arise in conducting overviews

**Table 6:** Data extraction

Step	Sub-step	Methods/approaches	Sources ▪ Examples
1.0 Plan the data elements to extract			
	1.1 Determine the data to extract on the characteristics of SRs^a^		Becker 2008 [1]; Caird 2015 [31]; JBI 2015 [40, 41]; Li 2012 [44]; Ryan 2009 [53, 54]
	1.2 Determine the data required to assess which SRs address the overview question and allow assessment of the overlap across SRs^a^		Smith 2011 [57]
	1.3 Determine data to extract about the results from the SRs for each relevant primary outcome		
		1.3.1 Extract M-A results	Becker 2008 [1]; Caird 2015 [31]; Hartling 2012 [35]; Smith 2011 [57]
		1.3.2 Extract numeric trial results	Thomson 2013 [59]
		1.3.3 Extract narrative results	Bolland 2014 [30]; JBI 2015 [40, 41]; Li 2012 [44]; Ryan 2009 [53, 54]
		1.3.4 Extract a combination of 1.3.1–1.3.3	
		1.3.5 Extract risk of bias assessment (overall assessment, or domain/item level data, or both) and certainty of the evidence	Becker 2008 [1]; Hartling 2012 [35]; JBI 2015 [40, 41]; Li 2012 [44]; Ryan 2009 [53, 54]
	1.4 Determine the data to extract from primary studies^a^		
		1.4.1 Extract numerical trial results	Caird 2015 [31]
		1.4.2 Extract data required to assess risk of bias for each domain or item	Hartling 2012 [35]
	1.5 Develop a data extraction form^a^		Becker 2008 [1]; Cooper 2012 [32]; Hartling 2012 [35]; JBI 2015 [40, 41]; Singh 2012 [56]
2.0 Plan the data extraction process			
	2.1 Determine the sources where data will be obtained from		
		2.1.1 SRs	Becker 2008 [1]; Bolland 2014 [30]; Caird 2015 [31]; CMIMG 2012 [4]; Hartling 2014 [37]; JBI 2015 [40, 41]; Pieper 2012 [6, 45]
		2.1.2 Primary studies	Caird 2015 [31]; Salanti 2011 [3]; Thomson 2013 [59]; Whitlock 2008 [48–52]
		2.1.3 Registry entries (for SRs and/or trials)	Inferred method
		2.1.4 A combination of the above	Caird 2015 [31]; Salanti 2011 [3]; Thomson 2013 [59]; Whitlock 2008 [48–52]
	2.2 Determine how overlapping information across SRs will be handled		
		2.2.1 Extract information from all SRs	Bolland 2014 [30]; Caird 2015 [31]; CMIMG 2012 [4]; Cooper 2012 [32]; Hartling 2014 [37]; JBI 2015 [40, 41]; Pieper 2014 [46]; White 2009 [48–52]
		2.2.2 Extract information from only one SR based on a priori eligibility criteria	Cooper 2012 [32]; CMIMG 2012 [4]; Foisy 2011 [34]; Hartling 2014 [37]; Pieper 2012 [6, 45]; Pieper 2014 [47]; Thomson 2013 [59] ▪ SR with the greatest number of trials (Cooper 2012 [32]) ▪ Most recent SR (Pieper 2014 [47]; Cooper 2012 [32])
	2.3 Determine how discrepant data across SRs will be handled in data extraction		
		2.3.1 Extract all data, recording discrepancies	Becker 2008 [1]; Bolland 2014 [30]; Caird 2015 [31]; Kovacs 2014 [42]; Pieper 2012 [6, 45]; Pieper 2014 [46]; Smith 2011 [57]; Thomson 2010 [58]
		2.3.2 Extract data from only one SR based on a priori eligibility criteria	Cooper 2012 [32]; Pieper 2014 [47] ▪ Most recent SR and SR of the highest quality (Pieper 2014 [47]) ▪ Highest quality SR (Cooper 2012 [32])
		2.3.3 Extract data element (e.g. effect estimates, quality assessments) from the SR which meets decision rule criteria	Bolland 2014 [30]; Cooper 2012 [32] ▪ SR that reports the most complete information on effect estimates (Bolland 2014 [30])
		2.3.4 Reconcile discrepancies through approaches outlined in 2.4	Bolland 2014 [30]; Caird 2015 [31]; Flodgren 2011 [33]; JBI 2015 [40, 41]; Salanti 2011 [3]; Thomson 2010 [58]; Whitlock 2008 [48–52]
	2.4 Determine additional steps to deal with missing data from SRs, or when there is variation in information reported across SRs		
		2.4.1 Retrieve reports of the primary studies	Bolland 2014 [30]; Caird 2015 [31]; CMIMG 2012 [4]; Flodgren 2011 [33]; Pieper 2012 [6, 45]; Pieper 2014 [47]; Salanti 2011 [3]; Thomson 2010 [58]; White 2009 [49–51]
		2.4.2 Contact SR or trial authors, or both, for missing info and/or clarification	Bolland 2014 [30]; Flodgren 2011 [33]; JBI 2015 [40, 41]; Whitlock 2008 [49–51]
		2.4.3 Search SR or trial registry entries for information	Inferred method
		2.4.4 A combination of the above approaches	Bolland 2014 [30]; Caird 2015 [31]; Salanti 2011 [3]; Thomson 2010 [58]; Whitlock 2008 [48–52]
		2.4.5 Do not take additional steps to deal with missing data or discrepancies	Becker 2008 [1]; Caird 2015 [31]; Foisy 2011 [34]; JBI 2015 [40, 41]
	2.5 Pilot the data extraction form^a^		Cooper 2012 [32]; JBI 2015 [40, 41]
	2.6 Determine the number of overview authors required to extract data^a^		
		2.6.1 Single, double, or more	Becker 2008 [1]; Bolland 2014 [30]; Hartling 2012 [35]; JBI 2015 [40, 41]; Li 2012 [44]; White 2009 [48–52]
		2.6.2 Data extraction versus data checking	Becker 2008 [1]; CMIMG 2012 [4]; Singh 2012 [56]; Whitlock 2008 [48–52] ▪ Evaluate a random sample of primary studies to ensure that data abstraction is accurate and reproducible (Whitlock 2008 [48–52])
	2.7 Determine if authors (co-)authored one or several of the reviews included in the overview, and if yes, plan safeguards to avoid bias in data extraction		Buchter 2015 [60] ▪ Overview authors do not extract data from their co-authored SRs

*CMIMG* Comparing Multiple Interventions Methods Group, *JBI* Joanna Briggs Institute, *M-A* meta-analysis, *SRs* systematic reviews

^a^Adaption of the step from SRs to overviews. No methods evaluation required, but special consideration needs to be given to unique issues that arise in conducting overviews

**Table 7:** Methods and approaches for addressing common scenarios unique to overviews

		Methods/approaches proposed in the literature^a^	
	Scenario for which authors need to plan	Eligibility criteria (Table 4)	Data extraction (Table 6)
1	Reviews include *overlapping* information and data (e.g. arising from inclusion of the same primary studies)	1.4.2 1.5 (1.5.1–1.5.4)	1.2 2.2 (2.2.1, 2.2.2)
2	Reviews report *discrepant* information and data^a^	1.4.2 1.6.2, 1.6.3	2.3 (2.3.1–2.3.4) 2.2.1, 2.2.2 2.4 (2.4.1–2.4.5)
3	Data are *missing* or reviews report *varying* information (e.g. information on risk of bias is missing or varies across primary studies because reviews use different tools)	1.6.2, 1.6.3	2.4 (2.4.1–2.4.5)
4	Reviews provide incomplete coverage of the overview question (e.g. missing comparisons, populations)	1.6.2, 1.6.3	1.2 2.1.2, 2.1.4 2.4
5	Reviews are not up-to-date	1.4.2 1.6.2, 1.6.3	2.1.2, 2.1.4
6	Review methods raise concerns about bias or quality	1.4.2 1.6.2, 1.6.3	1.2

^a^The methods/approaches could be used in combination

#### Characteristics of included articles

The characteristics of the included articles and the extent to which each described methods or approaches pertaining to the initial steps of an overview are indicated in Table 2. The majority of articles were published as full reports (*n* = 24/29; 83%). The most common type of article was one in which methods for overviews were described (*n* = 16/29; 55%), followed by articles that examined the methods used in a cross-section of overviews (*n* = 8/29; 28%), guidance documents (*n* = 4/29; 14%) and commentaries and editorials (n = 1/29; 3%). Methods for the specification of purpose, objectives and scope (*n* = 22); specification of eligibility criteria (*n* = 21); search methods (*n* = 18) and methods for data extraction (*n* = 17) were similarly mentioned or described. Relatively, few articles described methods across all of the initial steps in conducting an overview (*n* = 6).

#### Specification of purpose, objectives and scope

The two steps in the framework under ‘specification of purpose, objectives and scope’ were ‘determine stakeholder involvement in planning the overview (1.0)’ and ‘define the purpose, objectives and scope (2.0)’ (Table 3). In the following, we focus on the methods/approaches and options for the step ‘define the purpose, objectives and scope (2.0)’. Other methods/approaches are similar to those in planning a SR, but have been included in the framework for completeness.

We identified different purposes for undertaking an overview (2.1), with some of these purposes being ‘map the type and quantity of available evidence (2.1.1)’, ‘compare multiple interventions with the intent of drawing inferences about the comparative effectiveness of the interventions for the same condition (2.1.2)’ and ‘summarise the effects of an intervention across different conditions, populations, or problems (2.1.4)’. The latter borrows strength when there is sparse data for a single condition and a similar mechanism of action for the intervention is predicted across conditions. Options for confirming that an overview is the appropriate type of study for addressing the purpose and objectives (as compared with an intervention review or network meta-analysis) (2.2), included the ‘use of a decision tool (2.2.1)’ or ‘use other reasoning (2.2.2)’. A further identified sub-step was to ‘determine any constraints that will restrict the scope of the overview (2.3)’. Considerations arising from sub-steps 2.1–2.3 will influence whether an overview is conducted to address a narrow or broad question (2.4). This decision is then operationalised in the final identified sub-step ‘define the objectives using Population, Intervention, Comparison, Outcome (PICO) elements (or equivalent) to develop an answerable question (2.5)’.

#### Specification of eligibility criteria

The two steps in the framework under ‘specification of eligibility criteria’ were ‘plan the eligibility criteria (1.0)’ and ‘plan the study selection process (2.0)’ (Table 4). In the following, we focus on the step ‘plan the eligibility criteria (1.0)’, which covers methods that are key to dealing with common scenarios and challenges that arise in overviews (Table 7).

A unique decision in planning overviews is to ‘determine methodological eligibility criteria for SRs (1.4)’. Multiple criteria were identified, including approaches for selecting reviews that meet minimum quality criteria, or reviews that take a particular methodological approach (1.4.2). These criteria underpin many of the identified approaches for dealing with SRs with overlap in information and data (1.5). Overlap can arise when SRs with similar topics include one or more identical primary studies. One identified option was to include all SRs that meet the PICO criteria irrespective of overlap, that is, ignore overlap, note overlap, or deal with overlap using other methods (e.g. data extraction, synthesis) (1.5.1). However, other approaches aim to minimise overlap by specifying criteria to select one SR from multiple (1.5.2). These approaches include selecting one SR based on methodological criteria for SRs (see options in 1.4.2), selecting the most comprehensive SR, or excluding SRs that do not contain any unique primary studies (1.5.4). The latter approach may still result in inclusion of multiple overlapping SRs. An inherent complexity in using eligibility criteria to deal with overlap is that using single criteria can result in unintended loss of information through exclusion of important SRs (for example, the most recent SR could be excluded if only the highest quality SR is selected). An approach that overcomes this is to combine multiple criteria in an algorithm (1.5.3).

Another identified decision was whether to include additional primary studies (1.6). One option was to include primary studies only if pre-specified eligibility criteria are met (1.6.2). Circumstances that may prompt inclusion of primary studies are outlined in 1.6.2.

#### Search methods

The three steps in the framework under ‘search methods’ were ‘plan the types of sources to search (1.0)’, ‘plan the search strategy for retrieval of SRs (2.0)’, and ‘plan how primary studies will be retrieved, if eligibility criteria determines that primary studies should be included (3.0)’ (Table 5). Search methods for overviews largely parallel those used in a SR of primary studies. Unique considerations relate to the option to restrict searches to SR databases (1.1.1), the use of filters developed to retrieve SRs (2.1), and approaches to searching for additional primary studies.

If additional primary studies are eligible for the overview, authors will need to determine the sequence of searching for SRs and primary studies. The search for primary studies may be done in parallel with the search for SRs (3.1.1), or in sequence, searching first for SRs then for primary studies (3.1.2). The latter strategy focuses on retrieving primary studies where evidence is missing (i.e. where SRs are not up-to-date or where the SRs provide incomplete coverage of the overview question).

#### Data extraction

The two steps in the framework under ‘data extraction’ were ‘plan the data elements to extract (1.0)’ and ‘plan the data extraction process (2.0)’ (Table 6). We now highlight methods/approaches for dealing with these two steps, with a focus on methods for dealing with scenarios described in Table 7.

An identified sub-step in planning the data elements to extract (1.0) was determining the data to extract about the results from the SRs (1.3). For overviews, this will be driven by the purpose of the overview (e.g. whether the aim of the overview is to summarise results narratively from included SRs, or synthesise the results from component trials, or meta-analyses, from the included SRs). In addition to determining the data to extract about results from SRs, if the eligibility criteria of the overview include primary studies, then the data to extract from primary studies will also need to be determined (1.4).

A complexity that arises when undertaking an overview is the challenge of how to deal with overlapping (2.2) and discrepant (2.3) information and data across SRs. Identified options include extraction of information from all SRs, noting any discrepancies (2.2.1, 2.3.1), or extraction of data and information from only one SR (2.2.2, 2.3.2) based on pre-specified criteria, such as using the most recent SR, or the SR of the highest quality. Or, when there are discrepancies, different data elements (e.g. effect estimates, quality assessments) might be extracted from different SRs that meet certain decision rules (2.3.3), such as the SR that reports the most complete information on effect estimates. Methods for dealing with variation in the information reported and missing data are outlined in sub-step 2.4. In overviews, compared with SRs, there is additional complexity in resolving variation in information reported and missing data since there is an additional source of information (SRs in addition to primary studies).

#### Addressing common scenarios unique to overviews

Many of the identified methods were proposed to overcome common methodological challenges unique to overviews. Table 7 summarises these scenarios, showing methods that could be used to address each. While the literature reviewed often suggested a single method or step at which a scenario should be dealt with, Table 7 shows that there are multiple options, some of which can be combined.

### Stage II: identification and mapping of evaluations of methods

We found no studies that had evaluated methods in the steps of the framework for ‘specification of purpose, objectives and scope’, ‘specification of eligibility criteria’ and ‘data extraction’. Fifteen studies, published between 1998 and 2016, evaluated search filters for the retrieval of SRs (Table 8). One study [12], evaluated the performance of seven bibliographic databases to determine their coverage of SRs. This evaluation mapped to the option ‘select the types of databases to search’ (1.1.1) of the ‘search methods’ step of the framework (Table 5). Of the remaining 14 studies, two compared the performance of multiple published filters [13, 14], four developed new search filters and compared their performance against other published filters [15–18], and eight developed and evaluated new search filters (but without comparison with other published filters) [19–26]. These evaluations mapped to the option ‘select a published SR filter’ (2.1.1) of the ‘search methods’ step of the framework (Table 5).

**Table 8:** Characteristics of stage II evaluation of methods studies

First Author Year Title	Primary objective	Existence of a protocol	Study design	Health field the filter designed for	# of filters evaluated (# filters developed by the author)	Database (interfaces)	Technique to identify and/or create a gold standard	Sample size of the gold standard set or validation set (n)	Validation on a data set distinct from the derivation data	Performance measures used	Search dates for the gold standard or validation set	Name of filters evaluated (number of filters)
**Boluyt 2008** [13] Usefulness of systematic review search strategies in finding child health systematic reviews in MEDLINE.	Assess search filters for child health SRs in PubMed	NR	Comparative search filter evaluation	Child health	9	PubMed	Handsearching, Developed based on database searches	387	Yes	Sensitivity/recall, precision	Handsearch 1994, 1997, 2000, 2002, and 2004; DARE up to 2004, and year 2006	PubMed filter 2006 Shojania 2001 Boynton 1998 White 2001 (two) Montori 2005 (four)
**Boynton 1998** [15] Identifying systematic reviews in MEDLINE: developing an objective approach to search strategy design.	Evaluate propose a range of search strategies to identify SRs in MEDLINE	NR	Search filter evaluation, Comparative search filter evaluation	Medicine (general and internal)	15 (11)	MEDLINE (Ovid)	Handsearching	288	No	Sensitivity/recall, precision	1992 and 1995	Boynton 1998 (eleven) Hunt 1997 (two) CRD - Oxman 1994 (two)
**Eady 2008** [19] PsycINFO search strategies identified methodologically sound therapy studies and review articles for use by clinicians and researchers	Evaluate search strategies for finding SRs in PsycINFO	NR	Search filter evaluation	Psych.	N/A	PsycINFO	Handsearching	58	No	Sensitivity/recall, precision, specificity, accuracy	2000	Eady 2008
**Golder 2006** [20] Identifying systematic reviews of the adverse effects of health care interventions.	Identify SRs of adverse effects in two major databases	NR	Search filter evaluation	Adverse effects	N/A	DARE (CDSR and CRD)	Developed based on database searches	270	No	Sensitivity/recall, precision	1994 to 2005	Golder 2006
**Lee 2012** [16] An optimal search filter for retrieving systematic reviews and meta-analyses.	Develop and validate the health-evidence.ca SR filter and compare its performance to other filters	NR	Search filter evaluation, Comparative search filter evaluation	Public health	31 (3)	MEDLINE, EMBASE, and CINAHL	Handsearching, Developed based on database searches	219	Yes	Sensitivity/recall, precision, specificity NNR	2004/2005	health-evidence.ca SR filter - Lee 2012 (three) Montori [2005- four] Hunt 1997 (two) Shojania 2001 Boynton 1998 (two) BMJ Clin Evidence n.d. CRD Ciliska 2007 (four) SIGN n.d. Wilczynski 2007 (four) McKibbon 1998
**Montori 2005** [17] Optimal search strategies for retrieving systematic reviews from Medline: analytical survey.	Develop optimal search strategies in Medline for retrieving SRs	NR	Search filter evaluation, Comparative search filter evaluation	Medicinefamily practice, nursing, mental health	10 (4)	MEDLINE	Handsearching	753	Yes	Sensitivity/recall, specificity, precision	2000	Montori 2005 (four) White 2001 (three) Hunt 1997 (two) Shojania 2001
**Rathbone 2016** [12] A comparison of the performance of seven key bibliographic databases in identifying all relevant systematic reviews of interventions for hypertension.	Evaluate seven databases to determine their coverage of SRs of hypertension	NR	Comparative database evaluation	Hypertension	N/A	Cochrane, DARE, EMBASE, Epistemonikos, MEDLINE, PubMed, and TRIP	Developed based on database searches	440	N/A	Sensitivity/recall, precision	2003–2015	SR filters incorporated into the databases; MEDLINE used Montori 2005
**Shojania 2001** [21] Taking advantage of the explosion of systematic reviews: an efficient MEDLINE search strategy.	Evaluate a search strategy for identifying SRs	NR	Search filter evaluation	Treatment diagnosis, prognosis, causation, quality improvement, or economics	N/A	MEDLINE (PubMed)	Handsearching, Developed based on database searches	104	No	Sensitivity/recall, precision	1999–2000	PubMed n.d.
**White 2001** [18] A statistical approach to designing search filters to find systematic reviews: objectivity enhances accuracy.	Improve methods to derive a more objective search strategy to identify SRs in MEDLINE	NR	Search filter evaluation, Comparative search filter evaluation	Treatment diagnosis, prognosis, causation	7 (5)	MEDLINE (Ovid)	Handsearching journals	110	No	Sensitivity/recall, precision	1995 and 1997	White 2001 (five] Boynton 1998 CRD - Wolf 1996
**Wilczynski 2007** [22] EMBASE search strategies achieved high sensitivity and specificity for retrieving methodologically sound systematic reviews.	Develop search strategies that optimize the retrieval of SRs from EMBASE.	NR	Search filter evaluation	Internal medicinegeneral practice, mental health, nursing practice	N/A	MEDLINE	Handsearching journals	220	No	Sensitivity/recall, specificity, precision, accuracy	2000	Wilczynski 2007
**Wilczynski 2009** [23] Consistency and accuracy of indexing systematic review articles and meta-analyses in medline.	Determine the consistency and accuracy of indexing SRs and meta-analyses in MEDLINE	NR	Search filter evaluation	Medicine	N/A	MEDLINE	Developed based on database searches	NA	No	Sensitivity/recall, specificity, precision, accuracy	2000	Wilczynski 2009
**Wilczynski 2011** [24] Sensitive Clinical Queries retrieved relevant systematic reviews as well as primary studies: an analytic survey.	Determine how well the previously validated broad and narrow Clinical Queries retrieve SRs	NR	Search filter evaluation	Therapy, diagnosis prognosis, etiology	N/A	MEDLINE, EMBASE, CINAHL, and PsycINFO	Developed based on database searches	NA	No	Sensitivity/recall, specificity precision	2000	Wilczynski 2011
**Wong 2006** [14] Comparison of top-performing search strategies for detecting clinically sound treatment studies and systematic reviews in MEDLINE and EMBASE.	Compare sensitivity and specificity of search strategies for detecting reviews in MEDLINE and EMBASE	NR	Comparative search filter evaluation	Medicine	7	MEDLINE, EMBASE	Handsearching journals	753 in MEDLINE, 220 in EMBASE	N/A	Sensitivity/recall, specificity precision	2000	Montori 2005 (three) Wilczynski 2007 (four]
**Wong 2006** [26] Optimal CINAHL search strategies for identifying therapy studies and review articles.	Design optimal search strategies for locating review articles in CINAHL	NR	Search filter evaluation	Nursing and allied health	N/A	CINAHL	Handsearching journals	127	No	Sensitivity/recall, specificity precision, accuracy	2000	Wong 2006
**Zacks 1998** [25] Developing search strategies for detecting high quality reviews in a hypertext test collection.	Determine whether sensitive and specific search strategies exist to select SRs	NR	Search filter evaluation	Etiology, prognosis, therapy diagnosis	N/A	SWISH v.1.1.1	Developed based on database searches	209	No	Sensitivity/recall, specificity	Not reported	Zacks 1998

Sensitivity/recall is defined as the proportion of relevant reports correctly retrieved by the filter; Precision is the number of relevant reports retrieved divided by the total number of records retrieved by the filter; NNR is the inverse of the precision; Specificity is the proportion of irrelevant reports correctly not retrieved by the filter, Accuracy is the proportion of all reports that are correctly classified

*CDSR* Cochrane Database of Systematic Reviews, *CRD* Centre for Review and Dissemination, *n.d.* no date, *NNR* number needed to read, *N/A* not applicable, *NR* not reported, *PH* public health, *SRs* systematic reviews, *SWISH* Simple Web Indexing System for Humans

The filters were designed to retrieve SRs across a range of databases (CINAHL, DARE, EMBASE, PsycINFO, Epistemonikos, MEDLINE, Simple Web Indexing System for Humans (SWISH) and TRIP). Seven studies developed the gold standard by handsearching journals, three used a combination of handsearching journals and database searches and five used only database searches. The performance measures used included sensitivity/recall, specificity, precision, accuracy and the number needed to read. In terms of risk of bias, none of the evaluation studies referred to a study protocol or noted the existence of one, and only three validated their search filter on a data set distinct from the derivation set [13, 16, 17].

## Discussion

Despite the emergence of overviews as a common form of evidence synthesis, to date, there has been no comprehensive map of overview methods or the evidence underpinning these methods. We aimed to address this gap. A framework was developed for the initial steps in the conduct, interpretation and reporting of an overview (specification of the purpose, objectives and scope; specification of the eligibility criteria; search methods; and data extraction methods) with associated methods/approaches and options. The framework makes explicit large number of steps and methods that need to be considered when planning an overview and demonstrates some of the added complexity in an overview compared with a SR of primary studies. The framework also demonstrates that challenges in undertaking an overview, such as dealing with overlapping information across SRs, may be dealt with at different steps of the overview process (e.g. specification of eligibility criteria or data extraction). Fifteen evaluation studies were found in stage II, all of which mapped to the ‘search methods’ step of the framework. These studies either developed and evaluated a new search filter or compared the performance of existing search filters to retrieve SRs.

### What this study adds to guidance and knowledge about overview methods?

Our analysis aligns with findings of other recent reviews in identifying important gaps in guidance on the conduct of overviews [27, 28]. These gaps include patchy coverage of methods, wherein guidance covers selected options but not alternatives and insufficient description to operationalise many methods (Table 2). While others have concluded there is a lack of consensus over many methods [28], overviews serve many purposes, and different approaches are needed for different purposes. Recognising this, the framework attempts to capture the spectrum of options available to overview authors, providing a tool for systematic consideration of alternative approaches. We highlight the scenarios for which overview authors need to plan and identify methods proposed to tackle each scenario (Table 7). While these contributions help to address the patchy coverage of methods, the framework cannot address the lack of operational detail in current guidance. A forthcoming update of the Cochrane Handbook should help [28], but other guidance will be needed to cover the many methods not applicable to Cochrane overviews. For authors writing guidance, the framework could serve as a checklist to ensure comprehensive coverage of the methods proposed in the literature.

The lack of evaluation studies identified in stage II indicates that there is limited evidence to inform methods decision-making in overviews. For each of the steps in the framework, there is often a range of different methods to use, which could conceivably impact on the results and conclusions of the overview, their utility for decision-makers, and the time/resources required to complete the overview. This lack of evaluation of methods means [28] there may be inappropriate variability in the methods employed across overviews (as has been observed [6]). Further, overviews that seek to address the same research question, but which are undertaken using different methods, may reach discordant conclusions.

### How might the framework be used by overview authors and methodologists?

The framework may be useful to researchers conducting overviews and methodologists. As highlighted above, the framework is useful for making explicit the decisions overview authors need to make when planning an overview. Using the framework as a checklist to plan methods for dealing with common scenarios should lessen the challenges that arise when conducting an overview. Using the framework during protocol development may also lead to less post-hoc decision-making that can arise from not being aware of the decisions that need to be made before commencing the overview. Less post hoc decision-making may limit potential bias in the process of undertaking the overview. For overview methodologists, the provision of comparative options for each of the steps of the framework facilitates identification (and prioritisation) of methods evaluations that might be undertaken. For example, examining those steps where selection of a different option is hypothesised to importantly impact on the results and conclusions of the overview (discussed below under ‘Future research to refine and populate the framework and evidence map’).

### Strengths and limitations

To our knowledge, this is the first attempt to create a comprehensive framework of the many methods proposed for use in overviews. It is also the first study, of which we are aware, that has used evidence mapping in the context of methods research. A protocol of this investigation has been published [10] and any post hoc decisions have been documented. During our analysis, we developed an organising structure to group-related methods and used consistent language to synthesise the varied descriptions encountered in the literature. We also made inferences to ensure that where a clear alternative to a described method existed, it was captured in the framework. Both steps helped generate a more uniform and complete inventory of methods than would have been possible through simply collating methods as described.

Methods studies related to overviews are challenging to find other than in specialist methodology registers, such as the Cochrane Methodology Register and the Meth4ReSyn library, meaning that some methods articles may have been missed. We conducted reference checking and forward citation searching in three databases to minimise the number of missed articles. Further, we focused our search on locating articles that used the term ‘overview’ (or related terminology). However, methods that may be applicable to overviews, such as those used in clinical practice guidelines, may not have been located. We did not broaden our search, or specifically examine guidance documents for producing guidelines, to keep the project containable. Our analysis involved piecing together information spread across multiple sources, and ‘translating’ varied descriptions of methods into a common language. This process, and the many decisions involved in structuring our framework required considerable judgement. While the process led to a more complete and uniform description of methods than we identified in any other source, the subjective nature of this analysis means that other researchers may have made different decisions.

### Future research to refine and populate the framework and evidence map

Future research will involve seeking input on the framework from methodologists and researchers conducting overviews in terms of their face validity, that is, the structure of the framework and the comprehensiveness of the steps and identified methods. Hence, the framework will likely be refined and evolve over time. Further, as methods for overviews are evaluated, the evidence map can be further populated. While there is currently too little methods evaluation for a visual representation (or map) of the evidence to be useful, the framework provides the structure for creating this map. Some priority areas requiring evaluation, which we encourage methodologists to consider, relate to the decisions around eligibility and data extraction. For example, what is the effect of selecting one SR from multiple SRs addressing the same topic versus including all SRs? Outcomes of interest may include proximal measures, such as whether eligible primary studies or important data are missed. More distal measures include time taken to complete the overview, utility for decision-makers, and whether the findings and conclusions of the review change. Additionally, researchers could examine whether observed effects vary when different eligibility criteria are used to select one SR from a multiple. Similar questions can be posed about the effects of extracting data from one versus multiple SRs, from primary studies to SRs only, and so on. Evidence arising from these evaluations should lead to further refinement of the framework and, more importantly, empirical data about the trade-offs associated with alternative methodological approaches.

## Conclusions

A framework of methods for conducting, interpreting and reporting overviews of systematic reviews for the initial four steps of undertaking an overview was developed and populated. Studies evaluating methods for overviews were identified and mapped to the framework. Evaluation of methods allows us to make informed choices about the most appropriate methods to use. However, gaps in the evaluation of methods were found in the majority of steps. More evaluation of the methods used in overviews is needed. The results of this research are useful for identifying and prioritising methods research on overviews and provide a basis for the development of planning and reporting checklists.

## Additional files

Additional file 1: Search strategies. (DOCX 3.49 kb)

Additional file 2: Purposive search strategies. (DOCX 3.53 kb)

Additional file 3: Characteristics of excluded studies. (DOCX 5.65 kb)

Additional file 4: Table of reporting considerations. (DOCX 6.65 kb)

## Abbreviations

AHRQ’s EPC: Agency for Healthcare Research and Quality’s Evidence-based Practice Center
AMSTAR: A Measurement Tool to Assess Systematic Reviews
CDSR: Cochrane Database of Systematic Reviews
CMIMG: Comparing Multiple Interventions Methods Group
CRD: Centre for Reviews and Dissemination
JBI: Joanna Briggs Institute
M-A: Meta-analysis
N/A: Not applicable
NNR: Number needed to read
NR: Not reported
PH: Public Health
PICO: Population (P), intervention (I), comparison (C) and outcome (O)
PROSPERO: International Prospective Register of Systematic Reviews
RCT: randomised controlled trial
SRs: Systematic reviews
SWISH: Simple Web Indexing System for Humans
