# Supplementary material for: Toward a comprehensive evidence map of overview of systematic review methods: paper 1—purpose, eligibility, search and data extraction
Source: Syst Rev. 2017 Nov 21;6:231. doi: 10.1186/s13643-017-0617-1 (PMC5698938; doi:10.1186/s13643-017-0617-1)
Supplement: Supplementary file 1 — Search strategies. (DOCX 3.49 kb) [file 13643_2017_617_MOESM1_ESM.docx]

**Additional file 1**

**Search strategies**

Cochrane Methodology Register

[http://www.cochranelibrary.com](http://www.cochranelibrary.com/)

"overviews" or (overview* and review*) or metareview* or meta-review* or meta-synthesis or meta-syntheses or umbrella or "Cochrane overview*" or “review* of reviews” or "review* of systematic reviews"

Meth4ReSyn library

<http://www.citeulike.org/user/Meth4ReSyn>

overviews

or (overview && reviews)

or metareviews

or “meta-reviews”

or “meta-synthesis”

or “meta-syntheses”

or umbrella

or “Cochrane overview”

or “review of reviews”

or “reviews of reviews”

or “review of systematic reviews”

or “reviews of systematic reviews”

or (tag: review_of_reviews)

<http://www.citeulike.org/user/Meth4ReSyn/tag/review_of_reviews>

(note 1: if you put the term (tag:review_of_reviews) in the search engine, it will generate different results than if you click on the tag button on the right. I clicked on the tag on the right)

Cochrane Colloquium abstracts

[http://abstracts.cochrane.org](http://abstracts.cochrane.org/)

"overviews" or (overview* and review*) or metareview* or meta-review* or meta-synthesis or meta-syntheses or umbrella or "Cochrane overview*" or "review* of reviews" or "review* of systematic reviews"

"overview" or (review* and reviews*) or metaview* or data-reviews* or data-synthesis or data-synthesis or umbrella

"Cochrane overview*" or "review* of reviews" or "review* of systematic reviews"

Scientific Resource Center Methods library of the AHRQ Effective Health Care Program

<http://www.refworks.com/refworks2/?site=027181135918800000%2F57381342557464357%2FSRC+Methods+Library>

overviews

(overview && reviews)

metareviews

tag:“meta-reviews”

“meta-synthesis”

“meta-syntheses”

umbrella

“Cochrane overview”

“review of reviews”

“reviews of reviews”

“review of systematic reviews”

“reviews of systematic reviews”

(tag: systematic-reviews---reviews of)

<http://www.citeulike.org/user/EHCSRCMethodsLibrary/tag/systematic-reviews---reviews-of>

or (tag: systematic-reviews)

<http://www.citeulike.org/user/EHCSRCMethodsLibrary/tag/systematic-reviews>

Tag: systematic-reviews---meta-reviews

Tag: meta-reviews

<http://www.citeulike.org/user/SRCMethodsLibrary/tag/systematic-reviews---meta-reviews>

Tag: systematic reviews—overlapping (not downloaded just for future reference)

**Ovid MEDLINE (**Ovid MEDLINE(R) In-Process & Other Non-Indexed Citations, Ovid MEDLINE(R) Daily, Ovid MEDLINE(R) and Ovid OLDMEDLINE(R) 1946 to Present)

((overview$ or review) and reviews).ti. or "umbrella reviews".ti,ab. or (meta-reviews or metareviews).ti,ab. or metasyntheses.ti,ab. or "reviews of systematic reviews".ti,ab. or "reviews of reviews".ti,ab. or (overviews adj4 reviews).ab.
