# Supplementary material for: Toward a comprehensive evidence map of overview of systematic review methods: paper 1—purpose, eligibility, search and data extraction
Source: Syst Rev. 2017 Nov 21;6:231. doi: 10.1186/s13643-017-0617-1 (PMC5698938; doi:10.1186/s13643-017-0617-1)
Supplement: Supplementary file 2 — Purposive search strategies. (DOCX 3.53 kb) [file 13643_2017_617_MOESM2_ESM.docx]

**Additional file 2**

**Purposive search strategies**

Cochrane Methodology Register

[http://www.cochranelibrary.com](http://www.cochranelibrary.com/)

“search strategy” or “search filter” or [(retriev* or identif*) and search and reviews]

Meth4ReSyn library

<http://www.citeulike.org/user/Meth4ReSyn>

(tag: literature search)

<http://www.citeulike.org/user/Meth4ReSyn/tag/literature_search>

or +title: search strategy AND reviews

or +title: search strategy AND reviews

Cochrane Colloquium abstracts

[http://abstracts.cochrane.org](http://abstracts.cochrane.org/)

("search strategy*" and reviews) or ("search filter" and reviews) [limit 2015-2006]

Scientific Resource Center Methods library of the AHRQ Effective Health Care Program

(tag: searching)

<http://www.citeulike.org/user/EHCSRCMethodsLibrary/tag/searching>

or (tag: searching –filters)

<http://www.citeulike.org/user/EHCSRCMethodsLibrary/tag/searching---filters>

**or (tag: searching –systematic reviews)**

<http://www.citeulike.org/user/EHCSRCMethodsLibrary/tag/searching---systematic-reviews>

**Ovid MEDLINE (**Ovid MEDLINE(R) In-Process & Other Non-Indexed Citations, Ovid MEDLINE(R) Daily, Ovid MEDLINE(R) and Ovid OLDMEDLINE(R) 1946 to Present)

| 1. | ((search* and strateg*) or filter*).ti,ab. |
| --- | --- |
| 2. | (identif* or detect* or retriev*).ti,ab. |
| 3. | 1 and 2 |
| 4. | "systematic review*".ti,ab. |
| 5. | "Information Storage and Retrieval"/ |
| 6. | "Abstracting and Indexing as Topic"/ |
| 7. | 5 or 6 |
| 8. | 3 and 4 and 7 |

PubMed

(((search*[TIAB] AND (strateg*[TIAB] OR filter*[TIAB])) AND (identif*[TIAB] OR detect*[TIAB] OR retriev*[TIAB])) AND "systematic review*"[TIAB] AND ((information storage and retrieval[MH]) OR (abstracting and indexing[MH)))
