# Supplementary material for: Toward a comprehensive evidence map of overview of systematic review methods: paper 1—purpose, eligibility, search and data extraction
Source: Syst Rev. 2017 Nov 21;6:231. doi: 10.1186/s13643-017-0617-1 (PMC5698938; doi:10.1186/s13643-017-0617-1)
Supplement: Supplementary file 3 — Characteristics of excluded studies. (DOCX 5.65 kb) [file 13643_2017_617_MOESM3_ESM.docx]

**Additional file 3: Characteristics of excluded studies**

| **Citation** | **Reason for exclusion** |
| --- | --- |
| Adams 2000* [1] | Study not examining methods in a cross-section or cohort of overviews – does not discuss methods |
| Bekkering 2013* [2] | Study not examining methods in a cross-section or cohort of overviews – not a cohort of overviews |
| Bjordal 2008 [3] | Study not examining methods in a cross-section or cohort of overviews – does not discuss methods |
| Bramer 2013 [4] | Study not examining methods in a cross-section or cohort of overviews – not a cohort of overviews |
| Costa 2013* [5] | Study not examining methods in a cross-section or cohort of overviews – does not discuss methods |
| Delgado-Rodriguez 2006 [6] | Study not examining methods in a cross-section or cohort of overviews – not a cohort of overviews |
| Elliott 2004 [7] | Study not examining methods in a cross-section or cohort of overviews – not a cohort of overviews |
| Holt 2013 [8] | Study not examining methods in a cross-section or cohort of overviews – does not discuss methods |
| Hyde 2006* [9] | Study not examining methods in a cross-section or cohort of overviews – not a cohort of overviews |
| Jefferson 2000* [10] | Study not examining methods in a cross-section or cohort of overviews – not a cohort of overviews |
| Levin 2009 [11] | Study not examining methods in a cross-section or cohort of overviews – does not discuss methods |
| Lopez 2010* [12] | Study not examining methods in a cross-section or cohort of overviews |
| Martinez-Zapata 2011** [13] | Not a study that evaluated methods – not a cohort of overviews |
| Pantoja 2015* [14] | Study not examining methods in a cross-section or cohort of overviews |
| Rada 2013 [15] | Study not examining methods in a cross-section or cohort of overviews – not a cohort of overviews |
| Rojas 2011 [16] | Study not examining methods in a cross-section or cohort of overviews |
| Ryan 2005* [17] | Study not examining methods in a cross-section or cohort of overviews |
| Santaguida 2013 [18] | Study not examining methods in a cross-section or cohort of overviews – not a cohort of overviews |
| Tanjong-Ghogomu 2010* [19] | Study not examining methods in a cross-section or cohort of overviews |
| Thomson 2014 [20] | Study not examining methods in a cross-section or cohort of overviews – does not discuss methods |
| Wang 2012*[21] | Study not examining methods in a cross-section or cohort of overviews – not a cohort of overviews |

* Conference poster or presentation

** Stage II study

**References**

1. Adams C, Dooley, G., Jefferson, T., Lancaster, T.: Overviews - the way forward for the collaboration? In *Cochrane Colloquium*. Cape Town, South Africa; 2000.

2. Bekkering GE: Is there a potential of umbrella reviews to inform guideline development? In *Cochrane Colloquium*. Hannes K ed. Québec City, Canada; 2013.

3. Bjordal JM, Klovning A, Lopes-Martins RA, Roland PD, Joensen J, Slordal L: Overviews and systematic reviews on low back pain. *Ann Intern Med.* 2008, 148:789-790; author reply 791-782.

4. Bramer WM, Giustini D, Kramer BM, Anderson P: The comparative recall of Google Scholar versus PubMed in identical searches for biomedical systematic reviews: a review of searches used in systematic reviews. *Syst Rev.* 2013, 2:115.

5. Costa MB, Porfírio, G.J.M., Silva, V., Grande, A., Torres, M.F.S., Carvalho, M.R., Fioretti, B., Riera, R., Torloni, M.R.: Profile of overviews published by the Cochrane Library. In *Cochrane Colloquium*. Atallah A ed. Quebec City, Canada; 2013.

6. Delgado-Rodriguez M: Systematic reviews of meta-analyses: applications and limitations. In *J Epi & Comm Health*, vol. 60. pp. 90-92; 2006:90-92.

7. Elliott L, Crombie IK, Irvine L, Cantrell J, Taylor J: The effectiveness of public health nursing: the problems and solutions in carrying out a review of systematic reviews. *J Adv Nursing.* 2004, 45:117-125.

8. Holt RI: A review of reviews: a virtual issue. *Diabetes Obes Metab* 2013, 15:1-2.

9. Hyde C: Identifying systematic reviews on related topics: can we do more to help readers? In *Cochrane Colloquium*. Dublin, Ireland; 2006.

10. Jefferson T, Demicheli,, V.., Jefferson, T., Middleton, P., Wager, E.: An overview of the effects of peer review on the assessment of scientific submissions to journals and grant-giving bodies methodological issues. In *Symposium on Systematic Reviews: Beyond the Basics*. Oxford, UK; 2000.

11. Levin RF: Reviews, systematic reviews, overviews: "What's it all about, Cochrane"? *Res Theory Nurs Pract.* 2009, 23:256-258.

12. Lopez L, Grimes, D., Manion, C.: When it rains: synthesizing umbrella reviews of educational interventions In *Joint Colloquium of The Cochrane and Campbell Collaborations*. Keystone, USA; 2010.

13. Martinez-Zapata MJ, Rigau, D., Selva, A., Gich, I., Bonfill, X.: Applicability of R-AMSTAR instrument to appraise systematic reviews. In *Cochrane Colloquium*. Madrid, Spain; 2011.

14. Pantoja T, Opiyo, N., Ciaponni, A., Herrera, C., Lewin, S., Oxman, A., Paulsen, E., Rada, G., Wiysonge, C. : Strategies for improving health systems in low-income countries: lessons learnt from four overviews of systematic reviews of health systems interventions. In *Cochrane Colloquium; Vienna*. 2015

15. Rada G, Perez D, Capurro D: Epistemonikos: a free, relational, collaborative, multilingual database of health evidence. *Stud Health Technol Inform.* 2013, 192:486-490.

16. Rojas M, Lozano, J., Sola, I., Bonfill, X.: Incorporating the GRADE approach in overviews of systematic reviews: an example from an overview in neonatal respiratory care (P2A188). In *Cochrane Colloquium*. Madrid, Spain; 2011.

17. Ryan R, Hill, S.: Evidence overviews: prioritising areas for overviews of systematic reviews of interventions for effective communication with and participation by consumers In *Cochrane Colloquium*. Melbourne, Australia; 2005.

18. Santaguida PL, Keshavarz, H., Carlesso, L.C., Lomotan, M., Gross, A., MacDermid, J.C., Walton, D.M., ICON Working Group.: Suppl 4: A Description of the Methodology Used in an Overview of Reviews to Evaluate Evidence on the Treatment, Harms, Diagnosis/Classification, Prognosis and Outcomes Used in the Management of Neck Pain. *Open Orthopaed J.* 2013, 7:461.

19. Tanjong-Ghogomu E, Singh, J., Christensen, R., Wells, G., Suarez-Almazor, M., Buchbinder, R., Lopez-Olivo, A., Tugwell, P.: Overviews of reviews –methodological considerations of the Biologics for rheumatoid arthritis Cochrane overview. In *Cochrane Colloquium*. Keystone, USA; 2010.

20. Thomson D: Evidence synthesis in child health: overviews of reviews. *Evid Based Child Health* 2014, 9:1-2.

21. Wang X, Lindsley, K., Li, T.: Is there agreement in outcomes among Cochrane reviews to support ‘Overviews’ of reviews? a case study within the Cochrane Eyes and Vision Group (CEVG). . In *Cochrane Colloquium*. Auckland, New Zealand; 2012.
