# Supplementary material for: Toward a comprehensive evidence map of overview of systematic review methods: paper 1—purpose, eligibility, search and data extraction
Source: Syst Rev. 2017 Nov 21;6:231. doi: 10.1186/s13643-017-0617-1 (PMC5698938; doi:10.1186/s13643-017-0617-1)
Supplement: Supplementary file 4 — Table of reporting considerations. (DOCX 6.65 kb) [file 13643_2017_617_MOESM4_ESM.docx]

**Additional file 4: Table of reporting considerations**

**Table 1: Reporting considerations**

| **Step** | **Sub-step** | **Methods/approaches** | **Sources**  ▪ **Examples** |
| --- | --- | --- | --- |
| **1.0 Specification of purpose, objectives and scope** | | |  |
|  | 1.1 Identify the report as an overview of systematic reviews | | Hartling 2012 [1]; JBI 2015 [2, 3]; Li 2012 [4]; Pieper 2012 [5, 6]; Singh 2012 [7] |
|  | 1.2 Report the objectives and rationale with any deviations noted* | | Hartling 2012 [1]; Singh 2012 [7]; Smith 2011 [8] |
| **2.0 Specification of eligibility criteria** | | |  |
|  | 2.1 Report the PICO elements of the included SRs and note if there is a mismatch between the overview PICO and SR PICO | | Cooper 2012 [9]; Smith 2011 [8] |
|  | 2.2 Report the eligibility criteria and rationale (SRs and/or primary studies), irrespective of whether the criteria were pre-specified | | Bolland 2014 [10]; Caird 2015 [11]; Cooper 2012 [9]; Flodgren 2011 [12]; Pieper 2014 [13]; Whitlock 2008 [14-18] |
|  | 2.3 Report the outcome selection mechanism and rationale, irrespective of whether the mechanism was pre-specified | | Caird 2015 [11]; Cooper 2012 [9] |
|  | 2.4 Report other planned eligibility criteria methods with any deviations to those methods noted* | | Bolland 2014 [10]; Cooper 2012 [9]; Singh 2012 [7]; Robinson 2016 [14-18] |
| **3.0 Search methods** | | |  |
|  | 3.1 Report the search methods of the included SRs and any limitations of those searches | | Smith 2011 [8] |
|  | 3.2 Report the overview search approach used (i.e. sources searched, search sequence used [parallel or sequential search], dates searched, etc.) and the rationale, irrespective of whether the approach was pre-specified | | Becker 2008 [19]; Cooper 2012 [9]; CMIMG 2012 [20]; Hartling 2012 [1]; Hartling 2014 [21]; Ioannidis 2009 [22]; JBI 2015 [2, 3]; Li 2012 [4]; Pieper 2014 [13]; Robinson 2015 [14-18]; Singh 2012 [7]; Smith 2011 [8] |
|  | 3.3 Report other planned search methods with any deviations to those methods noted* | | Becker 2008 [19]; Cooper 2012 [9]; CMIMG 2012 [20]; Hartling 2012 [1]; Hartling 2014 [21]; Ioannidis 2009 [22]; JBI 2015 [2, 3]; Li 2012 [4]; Pieper 2014 [13]; Robinson 2015 [14-18]; Singh 2012 [7]; Smith 2011 [8] |
| **4.0 Data extraction** | | |  |
|  | 4.1 Report other planned data extraction methods with any deviations to those methods noted* | | Becker 2008 [19]; Bolland 2014 [10]; Caird 2015 [11]; Flodgren 2011 [12]; Kovacs 2014 [23]; Pieper 2012 [5, 6]; Pieper 2014 [24]; Salanti 2011 [25]; Smith 2011 [8]; Thomson 2010 [26]; Whitlock 2008 [14-18] |

CMIMG: Comparing Multiple Interventions Methods Group; JBI: Joanna Briggs Institute; PICOs: Population (P), intervention (I), comparison (C), outcome (O), and study design (s); SRs: systematic reviews.

* Adaption of the step from SRs to overviews. No methods evaluation required, but special consideration needs to be given to unique issues that arise in conducting overviews

References

1. Hartling L, Chisholm A, Thomson D, Dryden DM: A descriptive analysis of overviews of reviews published between 2000 and 2011. *PloS one* 2012, 7:e49667.

2. Aromataris E, Fernandez R, Godfrey CM, Holly C, Khalil H, Tungpunkom P: Summarizing systematic reviews: methodological development, conduct and reporting of an umbrella review approach. *Int J Evid Based Healthc* 2015, 13:132-140.

3. Joanna Briggs Institute: Methodology for JBI Umbrella Reviews. South Australia: The University of Adelaide; 2014.

4. Li LM, Tian JT, Tian H, Sun R, Liu Y, Yang K: Quality and transparency of overviews of systematic reviews. *J Evid‐Based Med* 2012, 5:166-173.

5. Pieper D, Buechter R, Jerinic P, Eikermann M: Overviews of reviews often have limited rigor: a systematic review. *Journal of Clinical Epidemiology* 2012, 65:1267-1273.

6. Büchter R, Pieper, D., Jerinic, P.: Overviews of systematic reviews often do not assess methodological quality of included reviews. Poster. In *19th Cochrane Colloquium;*, vol. Suppl. pp. 105-106. Madrid, Spain: Cochrane Database Syst Rev; 2011:105-106.

7. Singh JP: Development of the Metareview Assessment of Reporting Quality (MARQ) Checklist. *Revista Facultad de Medicina de la Universidad Nacional de Colombia* 2012, 60:325-332.

8. Smith V, Devane D, Begley CM, Clarke M: Methodology in conducting a systematic review of systematic reviews of healthcare interventions. *BMC medical research methodology* 2011, 11:15.

9. Cooper H, Koenka AC: The overview of reviews: unique challenges and opportunities when research syntheses are the principal elements of new integrative scholarship. *American Psychologist* 2012, 67:446-462.

10. Bolland MJ, Grey A, Reid IR: Differences in overlapping meta-analyses of vitamin D supplements and falls. *J Clin Endocrinol Metab* 2014, 99:4265-4272.

11. Caird J, Sutcliffe K, Kwan I, Dickson K, Thomas J: Mediating policy-relevant evidence at speed: are systematic reviews of systematic reviews a useful approach? *Evidence & Policy: A Journal of Research, Debate and Practice* 2015, 11:81-97.

12. Flodgren G, Shepperd, S., Eccles, M.: Challenges facing reviewers preparing overviews of reviews (P2A194). In *Cochrane Colloquium*. Madrid, Spain; 2011.

13. Pieper D, Antoine S, Neugebauer EA, Eikermann M: Up-to-dateness of reviews is often neglected in overviews: a systematic review. *J Clin Epidemiol* 2014, 67:1302-1308.

14. Robinson KA, Chou R, Berkman ND, Newberry SJ, Fu R, Hartling L, Dryden D, Butler M, Foisy M, Anderson J, et al: Integrating Bodies of Evidence: Existing Systematic Reviews and Primary Studies. In *Methods Guide for Effectiveness and Comparative Effectiveness Reviews*. Rockville (MD): Agency for Healthcare Research and Quality (US); 2008.

15. Robinson KA, Chou R, Berkman ND, Newberry SJ, Fu R, Hartling L, Dryden D, Butler M, Foisy M, Anderson J, et al: Twelve recommendations for integrating existing systematic reviews into new reviews: EPC guidance. *J Clin Epidemiol* 2016, 70:38-44.

16. Robinson KA, Whitlock EP, O'Neil ME, Anderson JK, Hartling L, Dryden DM, Butler M, Newberry SJ, McPheeters M, Berkman ND: Integration of existing systematic reviews. In *Research White Paper (Prepared by the Scientific Resource Center under Contract No 290-2012-00004-C)*. Rockville, MD: Agency for Healthcare Research and Quality; 2014.

17. White CM, Ip S, McPheeters MC, Tim S., Chou R, Lohr KN, Robinson K, McDonald K, Whitlock EP: Using existing systematic reviews to replace de novo processes in conducting comparative effectiveness reviews. In *Methods Guide for Comparative Effectiveness Reviews*. Rockville, MD: Agency for Healthcare Research and Quality; 2009.

18. Whitlock EP, Lin JS, Chou R, Shekelle P, Robinson KA: Using existing systematic reviews in complex systematic reviews. In *Annals of Internal Medicine*, vol. 148. pp. 776-782; 2008:776-782.

19. Becker LA, Oxman, A.D.: Overviews of reviews. Cochrane Handbook for Systematic Reviews of Interventions. . ((Eds.) JPTHSG ed. pp. 607-631. Hoboken, NJ: John Wiley & Sons; 2008:607-631.

20. CMIMG C: Review Type & Methodological Considerations -‐Background Paper for the First Part of the Paris CMIMG Discussion. 2012.

21. Hartling L, Vandermeer B, Fernandes RM: Systematic reviews, overviews of reviews and comparative effectiveness reviews: a discussion of approaches to knowledge synthesis. *Evidence-Based Child Health a Cochrane Review Journal* 2014, 9:486-494.

22. Ioannidis JPA: Integration of evidence from multiple meta-analyses: a primer on umbrella reviews, treatment networks and multiple treatments meta-analyses. *CMAJ* 2009, 181:488-493.

23. Kovacs FM, Urrutia G, Alarcon JD: Overviews'' should meet the methodological standards of systematic reviews. *Eur Spine J* 2014, 23:480.

24. Pieper D, Antoine S-L, Morfeld J-C, Mathes T, Eikermann M: Methodological approaches in conducting overviews: current state in HTA agencies. *Research Synthesis Methods* 2014, 5:187-199.

25. Salanti G, Becker L, Caldwell D, Churchill, R., Higgins J, Li T, Schmid C: Evolution of Cochrane Intervention Reviews and Overviews of Reviews to better accommodate comparisons among multiple interventions. In *Report from a meeting of the Cochrane Comparing Multiple Interventions Methods Groups*: Cochrane Comparing Multiple Interventions Methods Groups; 2011.

26. Thomson D, Russell K, Becker L, Klassen TP, Hartling L: The evolution of a new publication type: Steps and challenges of producing overviews of reviews. *Res Syn Method* 2010, 1:198-211.
